# Supplementary material for: Strong neuron-to-body coupling implies weak neuron-to-neuron coupling in motor cortex
Source: Nat Commun. 2019 Apr 5;10:1575. doi: 10.1038/s41467-019-09478-2 (PMC6450901; doi:10.1038/s41467-019-09478-2)
Supplement: Supplementary file 1 — Supplementary Information [file 41467_2019_9478_MOESM1_ESM.pdf]

Supplementary information for

Strong neuron-to-body coupling implies weak neuron-to-neuron coupling in motor cortex.

Kells et al.

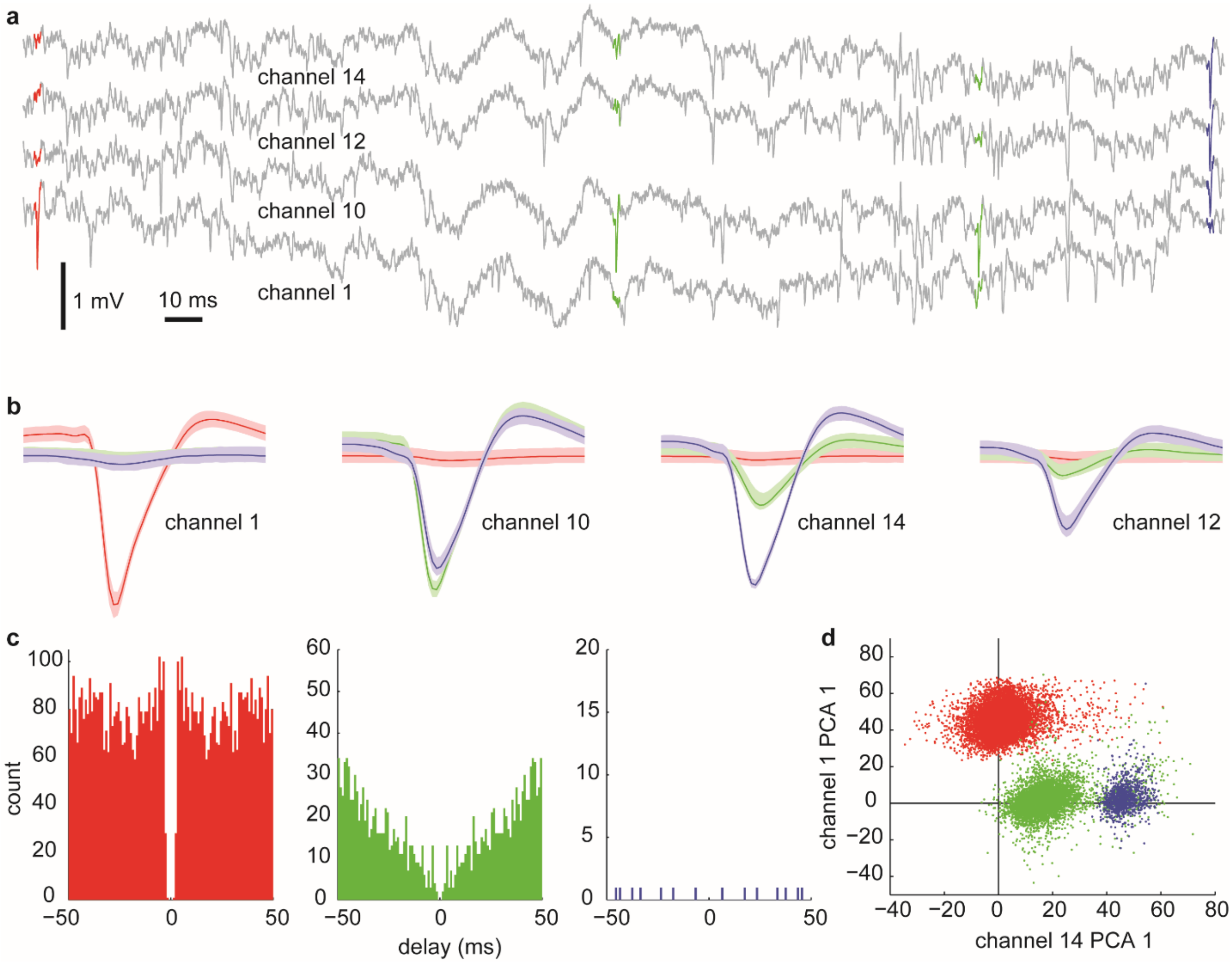

Supplementary Figure 1. Example demonstrating the quality of our spike sorting. A) Raw extracellular potentials recorded from a subset of 4 nearby electrodes. Time periods during which unit A (red), unit B (blue) and unit C (green) fired are color coded accordingly. B) Spike waveforms for units A, B, and C on the four electrodes. Solid line indicates median across many spikes; shaded area indicates quartiles. The differences in spike waveform across channels allows the spike sorting algorithm to distinguish the three units. C) Autocorrelograms for the spike times of units A, B, C. The gap for delays near zero delay is indicative of the refractory period expected of a single unit. D) In part, the spike sorting algorithm uses principle components of spike waveforms to determine that two units are different from each other. Each point represents one spike. The points are color coded according to which spike was assigned to which unit by the spike sorting algorithm. The well-separated clusters of points indicates that the units are easily distinguishable.

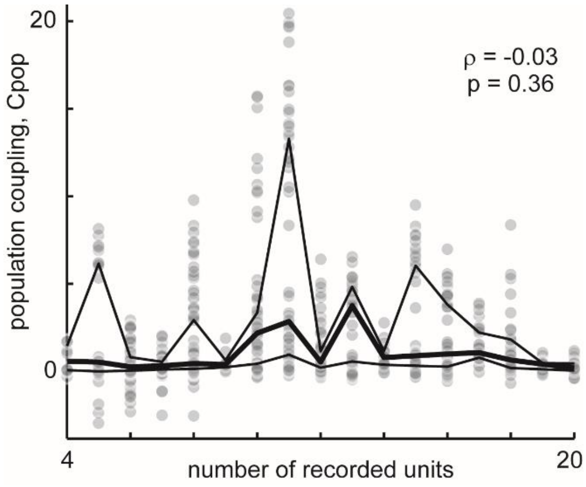

Supplementary Figure 2. The number of recorded single units ranged from 4 to 20 across all of our group 1 recording sessions (n=73). Population coupling was not significantly correlated with the number of recorded units.

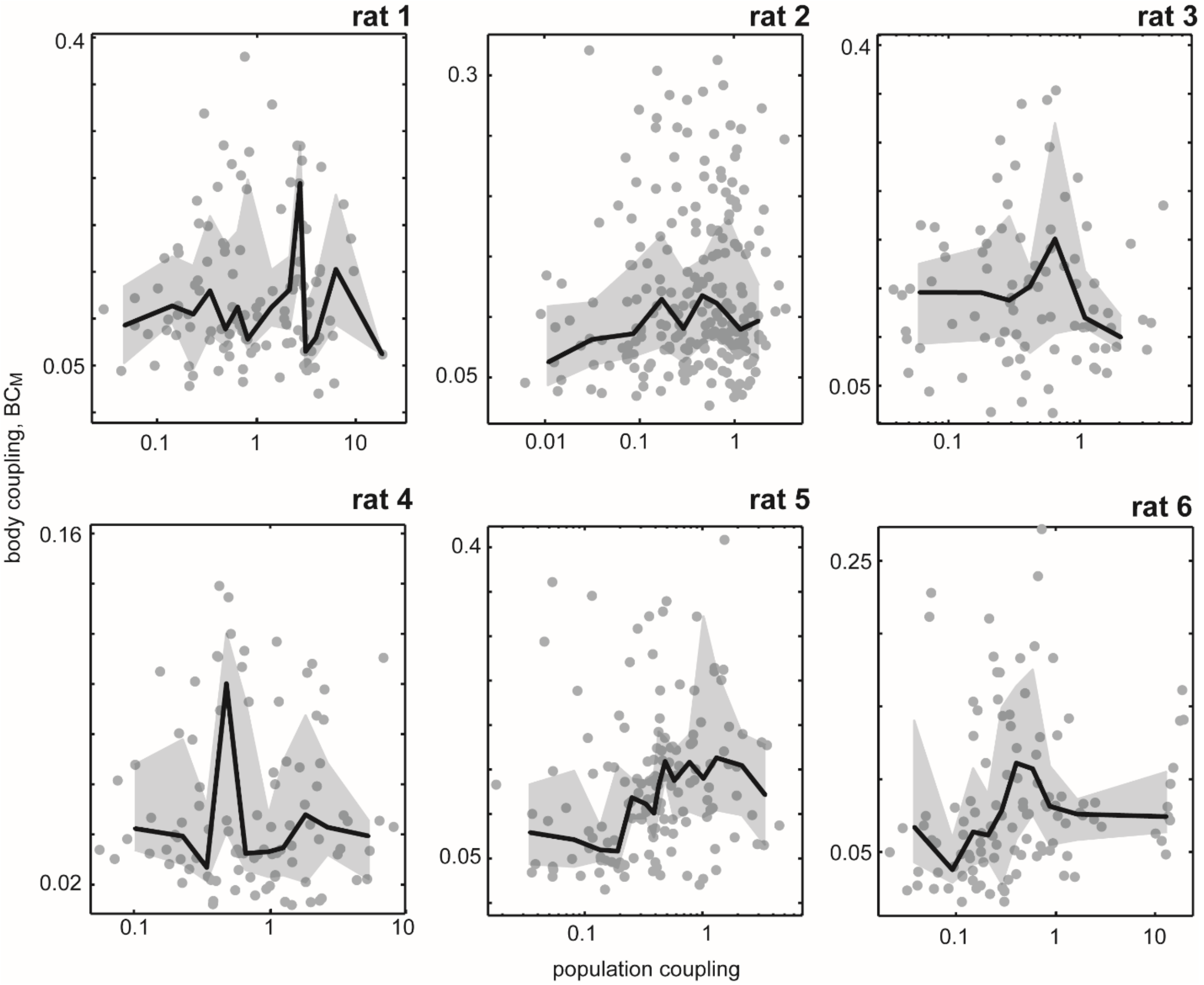

Supplementary Figure 3. In the results shown in Fig 4 in the main manuscript, we pooled units from all animals together. Here we show body coupling BC_M versus population coupling pooled only across recordings within each animal (one panel for each rat). Results with unaltered inhibition are shown.

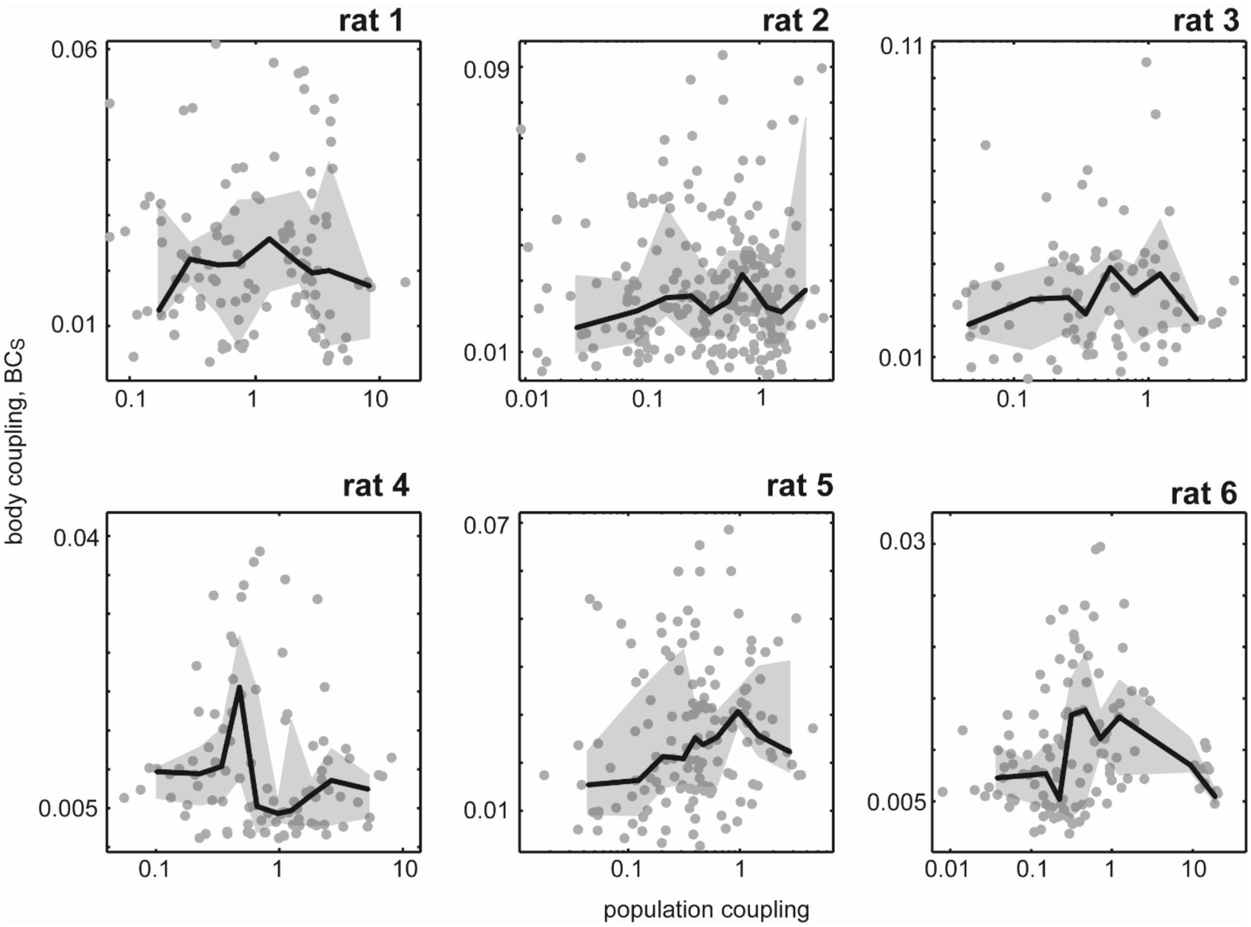

Supplementary Figure 4. In the results shown in Fig 5 in the main manuscript, we pooled units from all animals together. Here we show body coupling BC_S versus population coupling pooled only across recordings within one animal. Results with unaltered inhibition are shown.

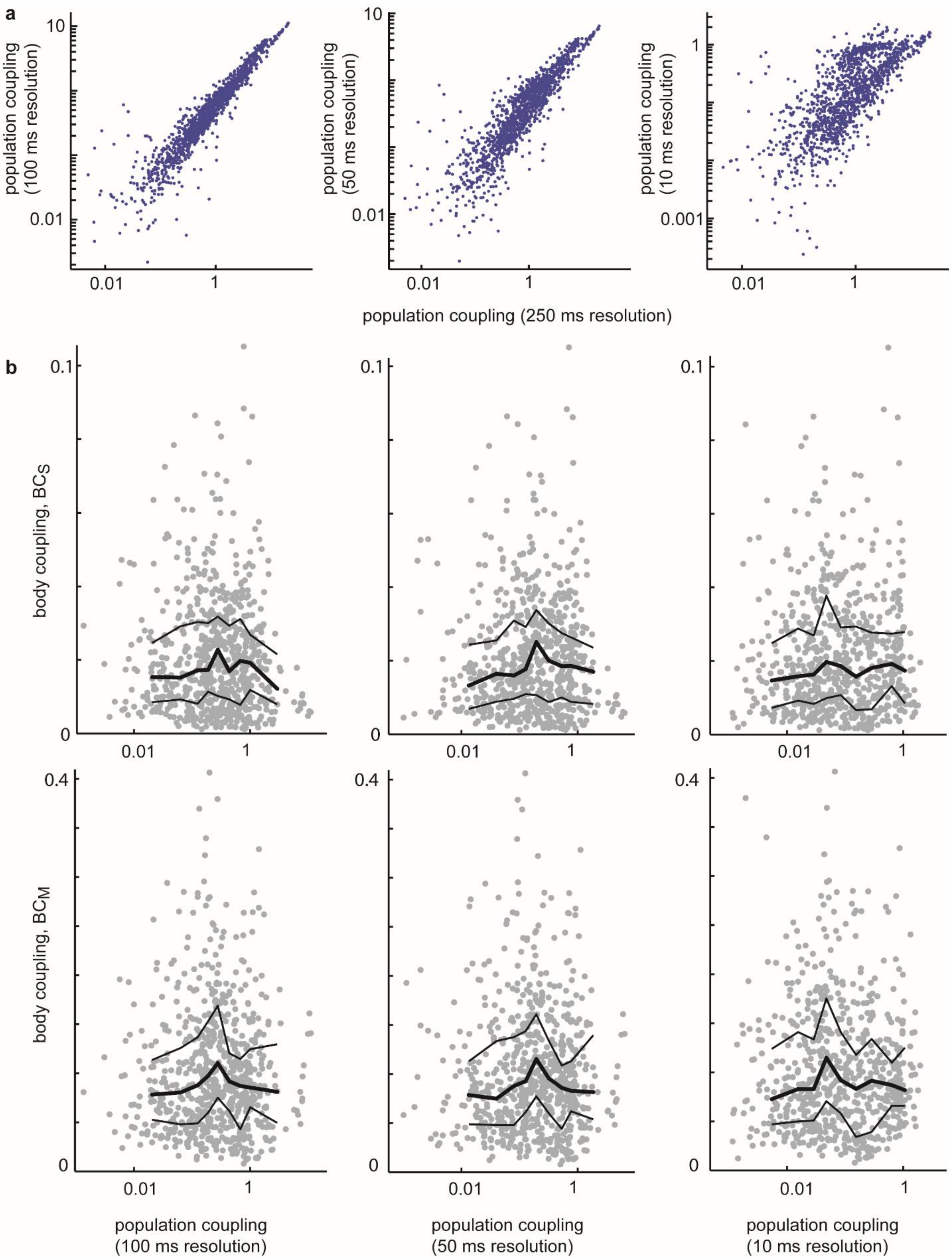
a
b
population coupling (100 ms resolution)
population coupling (50 ms resolution)
population coupling (10 ms resolution)
population coupling (250 ms resolution)
body coupling, BCS
body coupling, BCM
population coupling (100 ms resolution)
population coupling (50 ms resolution)
population coupling (10 ms resolution)

Supplementary Figure 5. Here we show that our results are robust to changes in the time resolution used to compute population coupling. **a.** We compare population coupling computed with different time resolutions (left panel: 100 ms, middle panel: 50 ms, right panel: 10 ms) versus that computed with 250 ms. We found that population coupling is highly correlated across these different timescales. **b.** For each time resolution, we plotted body coupling versus population coupling. We found that our primary findings (Figs 4d and 5d in the main manuscript) were robust to these changes in time resolution. We note that the peaked relationships were not statistically significant for BC_M at 50 and 10 ms resolution.

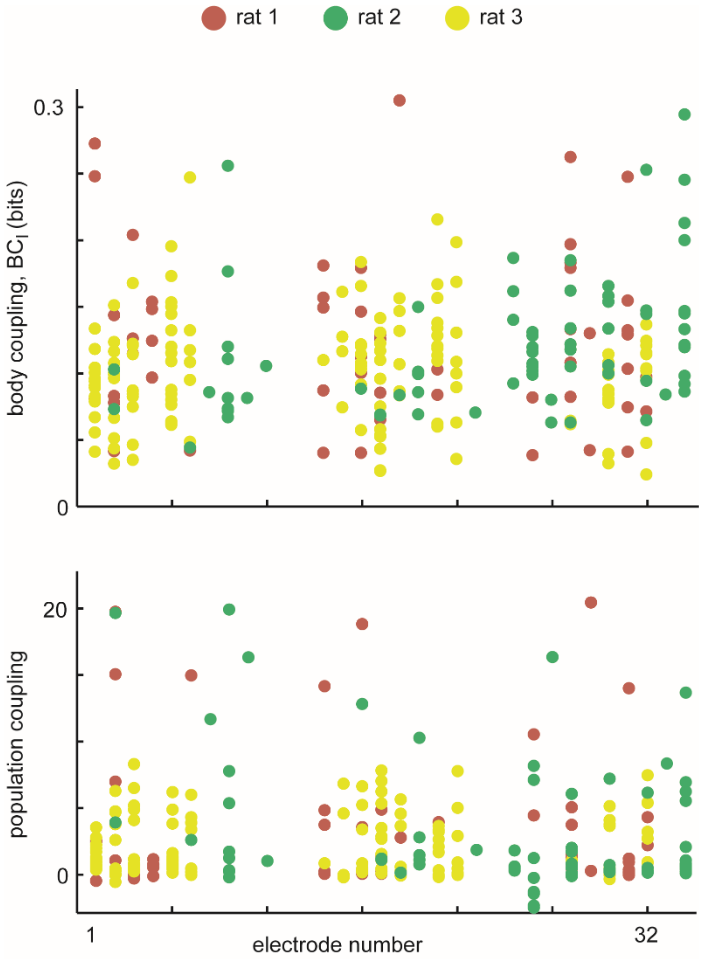

Supplementary Figure 6. Each dot represents one unit recorded on a particular electrode. Multiple dots for a single electrode represent different recording sessions, typically on different days. Color indicates rat. Typically, we observed a wide range of body coupling and population coupling on each electrode, which is consistent with two possibilities: 1) single units do not maintain fixed population coupling and body coupling on a day-to-day timescale, or 2) there are day-to-day changes in which units are recorded by a given electrode. Together with the fact that approximately 94% of units persist

across sessions on a given day, the former possibility is most likely.
